# Drug-induced Neuropsychiatric Adverse Events Using Post-Marketing Surveillance

**DOI:** 10.2174/1574884716666210215104540

**Published:** 2022-02-16

**Authors:** Tomohito Wakabayashi, Takahiro Nakatsuji, Hiroko Kambara, Iku Niinomi, Saki Oyama, Ayaka Inada, Sayaka Ueno, Mayako Uchida, Kazunori Iwanaga, Tatsuya Iida, Keiko Ho- sohata

**Affiliations:** 1 Education and Research Center for Clinical Pharmacy, Osaka University of Pharmaceutical Sciences, Takatsuki, Osaka, Japan

**Keywords:** Adverse events, spontaneous reporting system, reporting odds ratio, Japanese Adverse Drug Event Report (JADER) database

## Abstract

**Background:**

Several studies reported that abnormal behavior was noted in pediatric pa- tients receiving several drugs, including neuraminidase inhibitors (NIs). However, the information on drugs associated with abnormal behavior in a real-world setting remains limited. The purpose of this study was to clarify the drugs associated with abnormal behavior using a spontaneous reporting sys- tem database.

**Methods:**

We performed a retrospective pharmacovigilance disproportionality analysis using the Jap- anese Adverse Drug Event Report database. Adverse event reports submitted to the Pharmaceuticals and Medical Devices Agency were analyzed, and the reporting odds ratio at 95% confidence interval were calculated.

**Results:**

A total of 1,144 reports of abnormal behavior were identified. The signals were detected through the association of 4 neuraminidase inhibitors (oseltamivir, zanamivir, laninamivir, and peram- ivir) with the abnormal behaviour. These signals were stronger for oseltamivir than other neuramini- dase inhibitors. The signals were also detected for acetaminophen and montelukast.

**Conclusion:**

Our results should be able to raise physicians’ awareness of drugs associated with ab- normal behavior, but further investigation of these medications is warranted.

## 1 INTRODUCTION

Reported neuropsychiatric events are more common in children and generally occur in the early phase of the onset of influenza illness and the initiation of its treatment [1]. After categorizing events according to the International Classification of Disease, 9th revision (ICD-9) codes, ab- normal behavior comprises the largest category of such events [1]. Abnormal behavior caused by medicines in pedi- atric patients is a public health concern throughout the world [2, 3]. Several studies reported that such abnormal behavior is mostly associated with neuraminidase inhibitors (NIs) [4], however,information on drugs related to abnormal behavior in a real-world setting remains limited.

Recently, spontaneous reporting systems have been used as a crucial method of post-marketing drug safety surveil- lance for the detection of adverse drug events [5, 6]. The

Japanese Adverse Drug Event Report (JADER) database is a largely published database managed by the Pharmaceuti- cals and Medical Devices Agency (PMDA) for pharma- covigilance [7-11]. The objective of this study was to assess drugs associated with abnormal behavior in pediatric pa- tients using the JADER database.

## 2 METHODS

We used the JADER database as a spontaneous reporting database [7, 12]. The JADER database is freely obtainable from the website of the PMDA (http://www.pmda.go.jp/safety/info-services/drugs/adr-info/suspected-adr/0003.html), and we accessed the dataset of adverse event reports submit- ted between April 2004 and January 2017. It includes 4 ta- bles: patient demographic information, drug information, adverse events, and medical history. Physicians are present- ed to be the most frequent source of reports, while the re-maining reporters include pharmacists, other healthcare pro- fessionals, and patients.

In each case, the contribution of the medication to ad- verse events is classified into three categories: “suspected

medicine,” “concomitant medicine,” and “interaction medi- cine.” A “suspected medicine” is defined as a pharmaceuti- cal product suspected of causing an adverse event. When the reporter suspects a drug-drug interaction, he/she reports it as an “interaction medicine.” A “concomitant medicine” is defined as another pharmaceutical product used at the time of the adverse reaction. In this study, we only extracted cas- es that were classified as “suspected medicine.”

Next, we defined “pediatric patients” as the following age codes: “under 10s,” “10s,” and “pediatric” (157,314 reports). We compiled a cross-tabulation table based on two classifications: the presence or absence of the adverse event and the suspected medicine. Then, we calculated the reporting odds ratio (ROR) with the following formula:


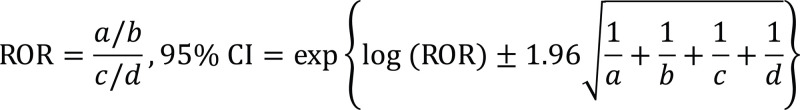


*a*: the number of patients developing the targeted event when they received the targeted drug

*b*: the number of patients developing non-targeted adverse events when they received the targeted drug

*c*: the number of patients developing the targeted event when they received non-targeted drugs

*d*: the number of patients developing non-target adverse events when they received non-targeted drugs.

In general, ROR is used with the spontaneous reporting database as an index of the relative risk of drug-associated adverse events. A signal was considered to be present when the lower limit of the 95% confidence intervals (CI) of the ROR was > 1.

In this database, age, height, and weight information are indicated as follows: age in decades, height in centimetres, and weight in kilograms. These data are not given as contin- uous variables because of privacy considerations, so we could not conduct multiple analyses using them. All anal- yses were conducted using JMP Pro 12 (SAS Institute Inc., Cary, NC, USA).

## 3 RESULTS

A total number of 157,314 reports on pediatric patients were used. Neuropsychiatric adverse events are presented in (Table **1**). Of those, abnormal behavior was most reported (1,144 reports) during the study period (Table **1**).

Next, we focused on abnormal behavior for further analysis. (Table **2**) shows the top 10 drugs associated with abnormal behavior. The most frequently reported drugs were oseltamivir phosphate (n = 545), followed by zanamivir hydrate (n = 320), laninamivir octanoate hydrate (n = 38), acetaminophen (n = 35), levetiracetam (n = 19), peramivir hydrate (n = 10), montelukast sodium (n = 9), lamotrigine (n = 9), d-chlorpheniramine maleate (n = 8), and amantadine hydrochloride (n = 8) and the signals were detected in these drugs without lamotrigine (ROR: 0.57; 95% CI: 0.3 - 1.1). The signal scores for oseltamivir phosphate (ROR: 139, 95% CI: 121 - 158), zanamivir hydrate (ROR: 64.7, 95% CI: 56 - 74.7), laninamivir octanoate hydrate (ROR: 63.1, 95% CI: 42.8 - 92.9), gemcitabine hydrochloride (ROR: 10.5, 95% CI: 8.96 - 12.2), and cyclosporine (ROR: 8.70, 95% CI: 7.67 - 9.86) were noteworthy. Of note, signals of acetaminophen and montelukast sodium (ROR: 4.74; 95% CI: 3.37 - 6.67) for abnormal behavior were detected.

## 4 DISCUSSION

In the present study, we identified 1,144 reports of ab- normal behavior in pediatric patients using the JADER spontaneous reporting system. The most frequently report- ed drugs associated with abnormal behavior were oselta- mivir phosphate, followed by zanamivir hydrate, la- ninamivir octanoate hydrate, acetaminophen, levetirace- tam, peramivir hydrate, montelukast sodium, lamotrigine, *d*-chlorphenira- mine maleate, and amantadine hydrochlo- ride. To the best of our knowledge, this is the first study to show the association of abnormal behavior with aceta- minophen and montelukast sodium in a real-world setting using a spontaneous database.

In our results, NIs such as oseltamivir phosphate, zanamivir hydrate, laninamivir octanoate hydrate, and per- amivir hydrate were associated with abnormal behavior,

which is consistent with clinical [13] and experimental [14] studies. On the other hand, other clinical studies reported that abnormal behavior occurred with the onset of influenza in children, irrespective of oseltamivir treatment [15-17]. A large prospective cohort study in children and adolescents with influenza showed that abnormal behavior was more likely to develop up to approximately 30 times during the period between the initial intake of oseltamivir and its Tmax (*i.e*., the period when the blood concentration of oseltamivir is likely to increase sharply), and this period overlapped with the early period of influenza where high fever was ob- served [18]. Therefore, the possibility could not be denied that abnormal behavior was induced by influenza itself.

In this study, acetaminophen was associated with ab- normal behavior. This is in agreement with several reports that acetaminophen may have some association with behav- ioral problems [19, 20]. Another report from the National Database of Electronic Medical Claims showed that aceta- minophen alone or co-administered with NI do not raise the risk of abnormal behavior in influenza patients. Further studies will be needed to clarify the situation.

The WHO Global database VigiBase^®^ reported that the reporting odds ratio of neuropsychiatric symptoms, such as suicidal ideation and abnormal behavior, is highest in chil- dren under 19 years old who were treated with montelukast [21]. A systematic review of adverse drug events showed that leukotriene antagonists are associated with neuro- psychiatric system disorders [22]. Moreover, a retrospective investigation of adverse effects in asthma patients treated only with leukotriene receptor antagonists showed that among 1,024 patients, 41 showed adverse effects and 24 of the 41 had neuropsychiatric symptoms [23]. These neuro- psychiatric symptoms included hyperactivity, hypersomnia and overnight phobia, and when the drug was discontinued, these adverse effects disappeared completely [23]. In the database of EudraVigilance, montelukast has been reported to be responsible for 2,210 psychotic adverse events in chil- dren with asthma [24]. In one case report, a 56-year-old man

diagnosed with major depressive disorder developed neuro- psychiatric symptoms, such as anxiety and insomnia, when he took fish oil supplements and montelukast [25]. When both were discontinued, the symptoms mostly disappeared [25]. These reports suggest that some factors may cause neuropsychiatric symptoms. It has been reported that monte- lukast causes neuropsychiatric symptoms by inhibiting the production of neurotransmitters, such as serotonin and nora- drenaline [21]. Because fish oil supplements include precur- sors of eicosanoids, such as leukotriene, it is considered that neuropsychiatric symptoms may be associated with lipid mediators [25]. However, montelukast may not be associat- ed with neuropsychiatric symptoms because it is not ex- pressed in leukotriene receptors of the brain [21]. Since asthma is associated with neuropsychiatric symptoms, such as depression and suicidality, it is considered that neuropsy- chiatric symptoms are associated not with montelukast but with the exposure to aeroallergens causing asthma [21, 26]. Consequently, neuropsychiatric symptoms may be caused by montelukast, lipid mediators, or asthma [21, 25, 26].

This pharmacovigilance study using the JADER data- base had several limitations. Firstly, as in all pharmacovigi- lance studies, we were unable to calculate the true incidence rates, notably due to lack of the total number of patients receiving the drugs of interest [27]. Secondly, there could be underreporting of adverse events that may be due to certain drugs. Thirdly, ROR does not provide a robust indication of the signal strength. In spontaneous reporting systems, in- cluding JADER, there are no control populations, so ROR is different from the “odds ratio” that is commonly used in epidemiological studies. Moreover, ROR indicates an in- creased risk of adverse event reporting and not the risk of an adverse event itself. Forthly, the extent of actual exposure in the treated population is not available from the database. Finally, there might be other confounding factors related to the outcomes, but the present method did not provide de- tailed clinical information on the patients [28].

## CONCLUSION

This is the first study to show the association of abnor- mal behavior with acetaminophen and montelukast sodium in a real-world setting. Physicians should be alerted so that they can take precautions against the associated adverse events and potentially avoid them.

## Figures and Tables

**Table 1:** The number of annual reports of neuropsychiatric adverse events in pediatric patients.

**PT Code**	**PT**	**Number of Reports**
10061422	Abnormal behavior	1144
10001854	Altered state of consciousness	768
10012218	Delirium	229
10012260	Delusions	36
10012373	Depressed level of consciousness	495
10024855	Loss of consciousness	902
10033664	Panic attack	3
10061910	Parasomnia	0

PT: Preferred terms in MedDRA.

**Table 2:** Most frequently reported drugs that induce abnormal behavior.

**Suspected Drug**	**n**	**ROR**	**95% CI**
Oseltamivir phosphate	545	139	121 - 158*
Zanamivir hydrate	320	64.7	56 - 74.7*
Laninamivir octanoate hydrate	38	63.1	42.8 - 92.9*
Acetaminophen	35	4.74	3.37 - 6.67*
Levetiracetam	19	2.57	1.63 - 4.06*
Peramivir hydrate	10	20	10.3 - 38.8*
Montelukast sodium	9	7.23	3.69 - 14.2*
Lamotrigine	9	0.57	0.3 - 1.1
*d*-chlorpheniramine maleate	8	14.1	6.79 - 29.2*
Amantadine hydrochloride	8	25	11.7 - 53.2*

*: signal detected; CI: confidence interval; ROR: reporting odds ratio.

## Data Availability

The data supporting the findings of the article is availa-ble in the PMDA at http://www.pmda.go.jp/safety/info-services/drugs/adr-info/suspected-adr/0003.html, reference number [7, 12].
